# Improved Shoot Regeneration, Salinity Tolerance and Reduced Fungal Susceptibility in Transgenic Tobacco Constitutively Expressing PR-10a Gene

**DOI:** 10.3389/fpls.2016.00217

**Published:** 2016-02-29

**Authors:** Parinita Agarwal, Mitali Dabi, Prashant More, Khantika Patel, Kalyanashis Jana, Pradeep K. Agarwal

**Affiliations:** ^1^Division of Wasteland Research, CSIR-Central Salt and Marine Chemicals Research Institute, Council of Scientific & Industrial ResearchBhavnagar, India; ^2^Academy of Scientific and Innovative Research, CSIR-Central Salt and Marine Chemicals Research Institute, Council of Scientific and Industrial ResearchBhavnagar, India

**Keywords:** cytokinin, docking, JcPR-10a, *Macrophomina phaseolina*, photosynthesis, salinity, tobacco, transgenics

## Abstract

Plants in ecosystems are simultaneously exposed to abiotic and biotic stresses, which restrict plant growth and development. The complex responses to these stresses are largely regulated by plant hormones, which in turn, orchestrate the different biochemical and molecular pathways to maneuver stress tolerance. The PR-10 protein family is reported to be involved in defense regulation, stress response and plant growth and development. The *JcPR-10a* overexpression resulted in increased number of shoot buds in tobacco (*Nicotiana tabacum*), which could be due to high cytokinin to auxin ratio in the transgenics. The docking analysis shows the binding of three BAP molecules at the active sites of JcPR-10a protein. *JcPR-10a* transgenics showed enhanced salt tolerance, as was evident by increased germination rate, shoot and root length, relative water content, proline, soluble sugar and amino acid content under salinity. Interestingly, the transgenics also showed enhanced endogenous cytokinin level as compared to WT, which, further increased with salinity. Exposure of gradual salinity resulted in increased stomatal conductance, water use efficiency, photosynthesis rate and reduced transpiration rate. Furthermore, the transgenics also showed enhanced resistance against *Macrophomina* fungus. Thus, JcPR-10a might be working in co-ordination with cytokinin signaling in mitigating the stress induced damage by regulating different stress signaling pathways, leading to enhanced stress tolerance.

## Introduction

Plants being sessile are strongly affected by climatic changes, pathogenic attack and these stresses represent a primary cause of crop-loss worldwide. These factors cause metabolic toxicity, membrane disorganization, closure of stomata, decreased photosynthetic activity, generation of reactive oxygen species (ROS) and altered nutrient acquisition (Hasegawa et al., 2000). The plants responsiveness and adaptability to these stresses result from the constant re-adjustment at physiological, biochemical and molecular level during their entire lifecycle.

Pathogenesis related-10 proteins are small proteins with cytosolic localization, conserved three dimensional structures, single intron and in some plant families the position is also conserved at 62 amino acid (Hoffmann-Sommergruber et al., 1997). These proteins have a broad spectrum of roles in plants response to abiotic, biotic factors and also toward growth and development. The RNase activity, ligand binding activity, posttranslational modification (phosphorylation) and phytohormone signaling provide some information into the mechanism of the regulation of PR-10 proteins, however, the presence of isoforms makes it difficult to decipher its exact mode of function.

The induction of pathogenesis-related (PR) genes in response to different environmental cues and to pathogens and parasites qualifies their deployment as important gene for multiple stress tolerance (Liu and Ekramoddoullah, 2006; Agarwal and Agarwal, 2014). The involvement of phosphorylation/dephosphorylation events in its activation is interesting and provides unique and unbiased insights into the complexity of its regulation. The proteome analysis of a salinity-tolerant *Arachis hypogaea* L. callus cell lines revealed the presence of significantly elevated levels of PR-10 proteins in differentially phosphorylated states as compared to its sensitive counterpart (Jain et al., 2006). Studies on upstream region of different PR-10 genes indicate the presence of *cis*-acting elements for WRKY, RAVI, bZ1P, ERF, SEBF, and Pti4 transcription factors indicating the role of these transcription factors in regulating PR-10 gene. An important feature of PR-10 proteins is a large, Y-shaped hydrophobic cavity that could be responsible for the intracellular transport of apolar ligands, as diverse as fatty acids, flavonoids, cytokinins, or brassinosteroids. Slight modifications of the structure and shape of this cavity would allow binding of different ligands, that would cause PR-10 proteins to perform diverse roles in plant stress signaling and development (Lebel et al., 2010). Studies on the ligand binding property of PR-10 are gaining momentum with special emphasis on cytokinins. Cytokinins play a major role in a wide array of biological processes crucial to plant development (Mok and Mok, 2001), and also integrate different environmental cues (Argueso et al., 2009). A number of *Arabidopsis thaliana* genes involved with CK signaling pathways were differentially affected by various abiotic stresses (Argueso et al., 2009). The regulation of PR-10 protein also bears relationship in activating other PR-10 proteins; silencing of MtPR10-1 from *Medicago truncatula* induced other PR proteins and increased tolerance against infection with *Aphanomyces euteiches* (Colditz et al., 2007). Some specific function are also observed in PR-10 proteins, Hyp-1 encoding an enzyme for hypericin (HyH) biosynthesis shows 45% homology to Betv1 class allergens (Bais et al., 2003). The *AoPR1* gene is involved in phenylpropanoid pathway (Warner et al., 1994).

In our earlier work (Agarwal et al., 2013), we reported the cloning of an important *JcPR-10a* gene from *Jatropha curcas*, an important biofuel crop grown in the wastelands of India. Transcript expression of *JcPR-10a* was upregulated in response to NaCl, salicylic acid (SA), methyl jasmonate and *Macrophomina phaseolina*. Furthermore, JcPR-10a recombinant protein exhibited RNase and antifungal activity against *Macrophomina*. In present study, we show that overexpression of *JcPR-10a* gene in tobacco leads to enhanced salinity stress tolerance and resistance toward *M. phaseolina* by reducing ionic, oxidative stress and enhanced photosynthesis via increased cytokinin accumulation in transgenic plants.

## Materials and Methods

### Construction of Plant Transformation Vector and Tobacco Transformation

The open reading frame (ORF) of *JcPR-10a* cDNA (Agarwal et al., 2013), was PCR amplified using JcPR-10TF and JcPR-10TR primers (**Supplementary Table S1**) flanked with *Xho*I and *Xba*I restriction sites, respectively, and cloned in in *Xho*I/*Xba*I sites of pRT101vector (Topfer et al., 1987). Thereafter, expression cassette containing 35S: *JcPR-10a*: Poly A was cloned in the pCAMBIA 1301 at the *Hind*III site and mobilized into the *Agrobacterium* strain LBA4404. The *Agrobacterium* cells, harboring binary plasmid (**Supplementary Figure S1A**), were used to transform *Nicotiana tabacum* L. *cv.* Petit Havana leaf disks according to Horsch et al. (1985). The transgenic shoots were regenerated on Murashige and Skoog (1962) medium supplemented with 5 μM BAP (6-benzylaminopurine), 1 μM IAA (indole-3-acetic acid), hygromycin (20 mg/l) and cefotaxime (300 mg/l).

### Confirmation of Gene Integration in Tobacco Transgenics

The putative transgenic plants were confirmed for Glucuronidase (GUS) activity at T_0_ and T_1_ stage. Genomic DNA was isolated from different T_0_ lines by CTAB buffer (Doyle and Doyle, 1987) and used for confirming transgene integration by PCR with hygromycin phosphotransferase (*hptII*), GUS and gene-specific primers (**Supplementary Table S1**). The copy number of the transgene was determined by Real-time quantitative PCR performed in a CFX detection system (Bio-Rad, USA) with 1x Sso Advanced SYBR green supermix (Bio-Rad, USA). The purified genomic DNA was quantified (Epoch spectrophotometer, Biotek, India) and diluted to 1, 10, and 100 ng/μl concentration. The PCR reactions were carried out using 3.75 ng GUS primers or 7.5 ng *NRA* (nitrate reductase) gene primers (NCBI accession number X06134, **Supplementary Table S1**, Guo et al., 2010) in 20-μl. The NRA gene was used as negative control. The reaction conditions were as follows: 95°C for 5 min, 1 cycle and 95°C for 1 min, 55°C for 30 s, and 72°C for 30 s, 45 cycles. At the end of the PCR cycles, the products were put through a melt curve analysis to verify the specificity of PCR amplification. The amplified product was run on a 1.5% agarose gel to confirm expected size. The experiments were repeated twice independently and standard curves were plotted using threshold cycle (*C*t) value to determine reaction efficiencies. The efficiency values were put in the following formula given by Shepherd et al. (2009) to determine the copy number ratio of GUS to NRA:

$$Ratio\,\;(GUS:NRA)={\left\{1+{\left(E\right)}^{Ct}\right\}}_{GUS}/{\left\{1+{\left(E\right)}^{Ct}\right\}}_{NRA}$$

### Plant Stress Treatments

To analyze the effect of salinity at seed germination level, the seeds from T_0_ plants were germinated on MS medium supplemented with NaCl (0, 100, 200, and 300 mM) and germination percentage was scored 15 days after seed inoculation.

To analyze the stress tolerance of *JcPR-10a* overexpressing tobacco plants, the *N. tabacum* wild-type (WT) and transgenics were subjected to stress in ½ Hoagland hydroponic medium (Hoagland and Arnon, 1950), as well as under greenhouse conditions. Fifteen-days-old WT and hygromycin positive T_1_ transgenic seedlings were transferred to ½ Hoagland hydroponic medium for 45 days. The uniform sized plants were subjected to NaCl stress (0, 100, and 200 mM) for a period of 15 days. Thereafter, morphological [root length, shoot length, fresh weight (FW), dry weight (DW)], physiological [relative water content (RWC), membrane stability index (MSI), electrolyte leakage (EL) and ion content], and biochemical parameters [total amino acid (TAA), total soluble sugar (TSS) and proline], were recorded. The quantification of phytohormones and expression of *IPT-1* gene was also studied in these WT and transgenics. The leaf disks from *N. tabacum* WT and T_1_ transgenics were floated on ½ MS liquid medium alone (major and minor salts, control) or supplemented with different NaCl concentrations for 3 days and incubated under continuous white light at 25 ± 1°C.

The WT and hygromycin positive T_1_ transgenic plants were hardened and transferred in earthen pots in green house. After 3 weeks of growth, plants were subjected to gradual salt treatment with slight modification as in Shukla et al. (2015). Plants were subjected to 400 ml of 50, 100, 200, and 300 mM NaCl treatment at 4, 5, 6, and 7 weeks, respectively. The different gas exchange and chlorophyll fluorescence parameters were recorded in both stressed and non-stressed plants from 5 to 7 weeks and 14 days after removal of stress on growth restoration (8–9 weeks).

### Real-Time PCR Analysis of *IPT-1* Gene in T_1_ Transgenic Plants

For quantitative expression of *IPT-1* gene (NCBI accession number: JX040475) the cDNA was prepared from the stress-treated WT and T_1_ transgenic plants. Five micrograms of RNA was treated with DNaseI (Thermo Scientific) followed by first strand cDNA synthesis using Revert Aid cDNA synthesis kit (Thermo Scientific). The cDNA was diluted to 1:10 and used as a template for Real-time PCR analysis. Tobacco actin gene was used as internal control gene (Shukla et al., 2015). Real-time PCR was performed using *IPT-1* primers (**Supplementary Table S1**) with 1x Sso Advanced SYBR green supermix (Sigma–Aldrich) with following PCR reaction: initial denaturation at 94°C, 2 min for 1 cycle; 94°C, 1 min, 55°C, 1 min and 72°C, 1 min for 45 cycles. The specificity of PCR amplification was checked at the end of the PCR cycles, by melt curve analysis. Each reaction was replicated three times and relative-expression was determined using Livak method (Livak and Schmittgen, 2001).

### Extraction and Quantification of Plant Growth Regulators from Tobacco Leaves

The plant growth regulators (PGRs) were extracted according to Pan et al. (2010) from tobacco leaf tissue exposed to different treatments and from their corresponding control seedlings (as mentioned earlier). The plant tissue was ground with liquid nitrogen and 50 mg of powdered tissue was transferred into 2 ml Eppendorf tube. The samples for plant growth hormone indole-3-acetic acid (IAA), SA and zeatin determination were prepared in triplicates. BAP and DHB (2, 5- dihydroxybenzoic acid) were used as internal standards (1 μg/sample) for zeatin and SA, respectively. IPA (indole-3-propionic acid, 0.2 μg/sample) was added as an internal standard for IAA (Agarwal et al., 2016). PGRs were extracted with 500 μl extraction buffer containing 2-propanol, concentrated HCl and water (2:0.002:1) at 4°C for 30 min (Pan et al., 2010). For partitioning of PGRs, 1 ml of dichloromethane was added to each tube and mixed by inverting at 4°C for 30 min. The above mixtures were centrifuged at 13,000 *g* for 10 min at 4°C and bottom layer (900 μl) was transferred to fresh tube and vacuum concentrated. The resulted residue was re-dissolved in 50 μl of methanol and was taken for high-performance liquid chromatography (HPLC) analysis. To determine a linear range of each PGRs, standard curve using 0.05–10 μg/ml PGRs with internal standards of 10 μg/ml for SA, zeatin and 2 μg/ml IPA was prepared. The area ratio of standard and internal standard was plotted against concentration ratio of standard and internal standard. The concentration ratio of PGRs standards and internal standards was plotted against the HPLC peak areas of PGRs standards and internal standards.

The HPLC analysis was carried out employing SHIMADZU prominence instrument (Spincotech pvt. Ltd.) equipped with DAD detector and auto sampler. Injection volume for each sample was 50 μl throughout the analyses. Chromatographic separation was performed using Enable C18H (150 mm × 4.6 mm, 5 μm) column. Gradient elution was carried out according to Pan et al. (2010) with minor modifications. The HPLC gradient system consists of A: water with 0.1% formic acid and B: methanol with 0.1% formic acid. The gradient was started with 30% B with a hold of 2 min, increased linearly to 100% B in 2–20 min and again held for 2 min before finally increased linearly to 30% in 22–25 min. An equilibration time of 5 min was given at the end of each elution before starting the next analysis. The flow rate was maintained 0.8 ml/min and column heater temperature was set at 40°C. UV detection was carried out at 273, 305, and 254 nm wavelength for IAA, SA, and zeatin, respectively. Data analysis was performed using SHIMADZU LC solution software.

### Docking of 6-Benzylaminopurine to JcPR-10a Model Structure

Docking was performed using a grid based Autodock 4.2 program. The PR-10 protein pdb file generated by Phyre was considered for the docking study with BAP. Autodock utilizes Lamarckian Genetic Algorithm (LGA) to explore the grid space and performs energy evaluations on the position of the ligand with respect to the target energy grids. The grid box of 70-70-70 Å was used to close the protein and the drug in grid map preparation for the Autogrid simulation. In Autodock simulation, ligands explore six spatial degrees of freedom (i.e., rotation and translation) along with associated torsions, and the interaction energy is calculated at each step until global energy minimum is reached.

### Molecular Dynamic Simulation

Further, we have performed dynamic calculation using Gromacs-4.5.5 package. The total system of three BAP, JcPR-10a protein and water solvent was minimized, followed by 500 ps of NVT heating to 300 K and 500 ps of NPT equilibrium at 300 K. We have performed the equilibration under the NVT and NPT ensemble until all properties of interest have stabilized. For instance, in NVT, once the temperature is stabilized, the system is equilibrated under this ensemble and for the NPT, the pressure and temperature was stable before proceeding. Finally, a 5 ns molecular dynamics simulation has been carried out after equilibration.

### Physiological and Biochemical Analysis of Transgenic Plants in Response to Stress

The RWC, MSI, EL, proline content, TSSs, TAAs and ion content were measured as described in Shukla et al. (2012).

### *In Vivo* Localisation of O_2_^-^ and H_2_O_2_

For *in vivo* detection of O_2_^-^ and H_2_O_2_, the leaves were stained with nitro blue tetrazolium (NBT) and 3, 3′- diaminobenzidine (DAB) as described by Shi et al. (2010). After incubation, the chlorophyll was bleached in absolute ethanol and visualized for the presence of the blue and brown spots, respectively, for O_2_^-^ and H_2_O_2_.

### Gas Exchange and Chlorophyll Fluorescence Measurement

Photosynthetic gas exchange measurements were performed on third leaf from the top of the plants grown in green house by open infrared gas analyser (IRGA, Model Li-6400XT, Li-Cor, USA) as reported in Shukla et al. (2015).

### *In Vitro* Antifungal Activity of JcPR-10a Transgenics

Leaves from *in vitro* grown plants on MS basal medium were used for *in vitro* leaf and leaf extract assay. The second youngest and fully expanded leaf from WT and transgenic lines (L1, L4, and L6) were used for leaf assay and SA quantitative analysis. The inoculation was done by using a ∼2 mm-diameter agar plug containing actively growing *M. phaseolina* from PDA plates. Each leaf was kept on 0.8% agar (with 100 μg/ml of carbenicillin) plate, and then the PDA plug with *M. phaseolina* were placed upside down on the mid vein region of the leaf to facilitate the spread of infection more effectively as the mid vein is responsible for supplying the nutrients to the entire leaf. The PDA plugs without *M. phaseolina* were used as control. The leaf surface was observed 3 dpi (days post inoculation) under stereomicroscope (Leica L2) and photographed at the same magnification.

For leaf extract assay, leaf tissues (1 g each) from WT and *JcPR-10a* transgenics (L1, L4, and L6) was grinded in 3 ml sodium phosphate buffer (pH 7.0) and incubated at room temperature for 30 min. The leaf extract was centrifuged at 13,000 rpm and the supernatant collected was filter sterilized (FS). The FS leaf extract (500, 1000, and 1500 μl) of WT and each transgenic was separately added to 100 ml PDA followed by pouring equal volume (20 ml) into five petriplates. The mycelia of *M. phaseolina* were inoculated on PDA petriplates supplemented with leaf extracts and incubated at 28°C for 72 h. The radial growth (cm) of black microsclerotia reproductive zone and total growth zone of the fungus was measured and photographed. The experiment was repeated three times with three replicates for each control and treatment.

### Statistical Analyses

Each experiment was repeated thrice and the mean values and standard deviations were calculated. Analysis of variance was calculated using Fishers Least Significant Difference (LSD) by Infostat software at *P* ≤ 0.05 to determine the significance of difference between the means of control and different stress treatments. Mean values of treatments that were significantly different from each other were indicated by different alphabets.

## Results

### Overexpression of *JcPR-10a* Showed Increased Regeneration Efficiency

#### T_0_ Transgenic Lines

**FIGURE 1:**
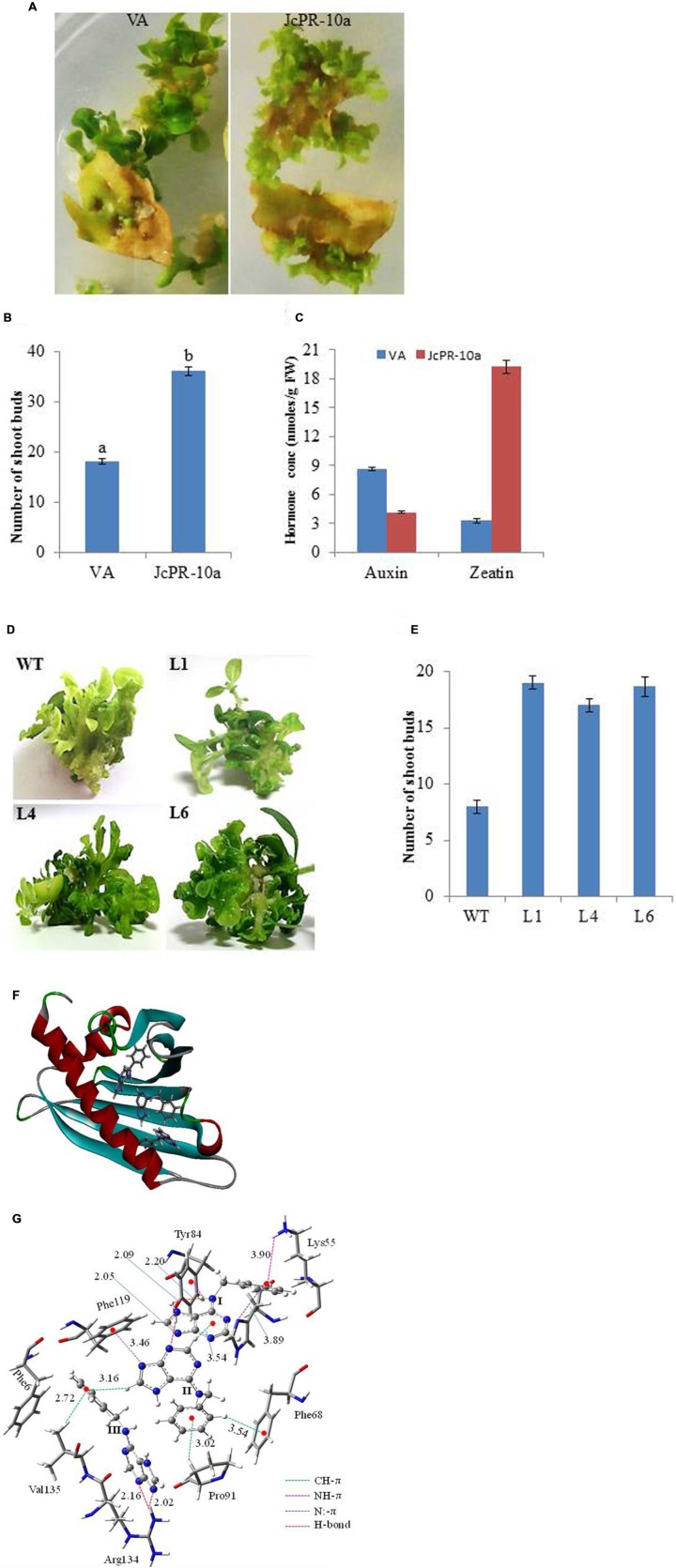
**(A)** Shoot bud induction on regeneration medium on explants transformed with VA (vector alone, transgenic plants transformed with pCAMBIA1301) and *JcPR-10a* gene. **(B)** Graph showing number of shoot buds induced on regeneration medium after 30 days of transformation. Data represent the mean ± SE of three biological repeats. **(C)** Auxin and cytokinin concentration in regenerated shoot buds. Data represent the mean ± SE of three biological repeats. **(D)** Regeneration potential of WT and transgenic (L1, L4, and L6) on MS medium supplemented with 1 μM BAP. **(E)** Graph showing number of shoot buds induced on MS medium supplemented with 1 μM BAP after 20 days of subculture. Data represent the mean ± SE of three biological repeats. **(F)** The BAP molecules at the active site of the JcPR-10a protein. **(G)** Non-covalent interactions between the active site amino acid residues and BAPs. Amino acid residues are represented in tube fashion and BAP in ball and stick fashion. (gray: carbon; blue: nitrogen; white: hydrogen; red: oxygen).The interaction distance is given in Å.

The leaf explants transformed with *JcPR-10a* gene showed higher number (36) of shoot buds on regeneration medium as compared to VA (18) after 30 days of culture (**Figures 1A,B**). The regenerated *JcPR-10a* transgenic plantlets showed an accumulation of 19.19 nmoles/g FW *in situ* zeatin and 4.13 nmoles/g FW *in situ* auxin (**Figure 1C**), showing an increase of 5.8- fold zeatin and decrease of 0.5-fold auxin concentration as compared to VA. To further study the extent of regeneration, the nodal segments of WT and transgenic lines (L1, L4, L6) were subcultured on MS medium supplemented with low BAP concentration (1 μM), interestingly, the transgenic lines showed higher number of well-developed shoots compared to WT (**Figures 1D,E**).

The putative transgenic lines selected on hygromycin- containing medium were subsequently verified by GUS analysis (**Supplementary Figure S1B**). Some plants showed full expression while others have scattered blue spots. The GUS positive transgenic lines showed amplification of GUS, *hptII* and *JcPR-10a* (483 bp) gene (**Supplementary Figures S1C–E**). Three selected transgenics were transferred to plastic pots for hardening and later transferred to earthen pots.

### Docking of BAP to JcPR-10a Protein Model

It was observed that three BAP molecules were stabilized by non-covalent interactions such as CH-π, NH-π, N:-π (lone pair of nitrogen and pi electron cloud) and hydrogen bonding interactions (Bernstein et al., 1995; Meyer et al., 2003; Jana and Ganguly, 2014) in JcPR-10a protein. The active site residues such as phenylalanine 68, tyrosine 84, phenylalanine 119, arginine 134, lysine 55, valine 135 and proline 91 amino acid residues played significant role to stabilize the BAP at the active site periphery. It was observed that purine moiety of the BAP (I) was stabilized by hydrogen bonding interaction with tyrosine 84 residue (2.09 and 2.20 Ǻ) and phenyl moiety by the NH-π (3.90 Ǻ) with lysine 55 amino acid residue, N:-π (3.89 Ǻ) between the nitrogen lone pair of histidine 70. It made the CH-π (3.54 Ǻ) interactions with another BAP molecule (II). The purine fragment of the BAP (II) formed N:-π (3.46 Ǻ) interactions with the phenylalanine 119 residues, whereas the phenyl moiety of BAP (II) was stabilized by CH-π interactions with the proline 91 and phenylalanine 68 residue (**Figure 1F**). The purine moiety of BAP (III) residue formed hydrogen bonding interactions (2.02 and 2.16 Ǻ) with the arginine 134 amino acid residue. The valine 135 amino acid residue made CH-π interaction with phenyl moiety of the BAP (III). The total system of three BAP, JcPR-10a protein and water solvent was equilibrated with 500 ps of NVT equilibrium at 300 K and 500 ps of NPT equilibrium at 300 K and a 5 ns MD (molecular dynamics) simulation after equilibration. The final MD geometry was considered for the free energy calculation using the gromacs which revealed the stability of the three BAP (-20.64 kJ/mol) molecules at the active site of the JcPR-10 protein. The structural orientations of the active site amino acid residues were similar to the docked structure (**Figure 1G**).

### Overexpression of *JcPR-10a* Confer Enhanced Tolerance to Salinity Stress

#### T_1_ Transgenic Lines

##### Analysis of T_1_ Transgenic Lines

The transgenics followed Mendelian segregation ratio of 3:1 Hyg^r^/Hyg^s^ (**Supplementary Table S2**) and seedlings were found GUS positive (**Supplementary Figure S1B**). Real-time PCR analysis revealed that L1, L4, L6 have GUS: NRA ratio between 0.25 and 0.9, thus confirming the single copy gene insertion (**Supplementary Figure S1F**). Further, T_1_ transgenic progeny was studied to establish the stress tolerance potential of tobacco transgenic overexpressing *JcPR-10a*. Several important growth parameters such as root length, shoot length, fresh weight, and dry weight of seedlings was measured as an indicator of salinity tolerance because changes in root and shoot growth are of potential importance in increasing stress tolerance.

**FIGURE 2:**
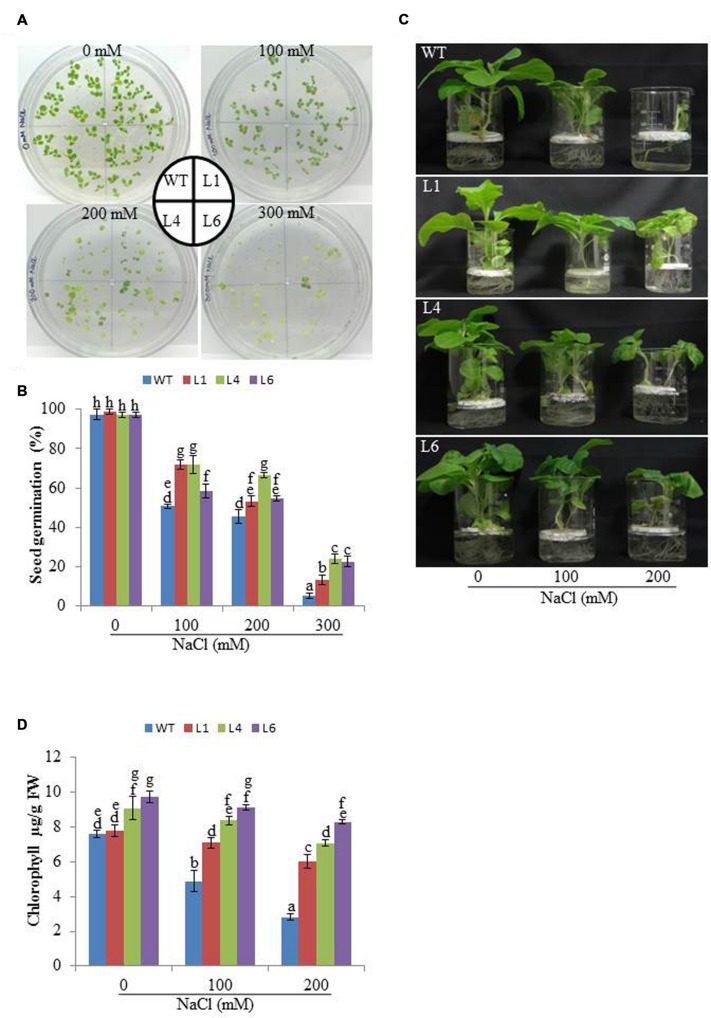
**(A)** Analysis of seed germination, **(B)** Percentage seed germination, **(C)** Morphological analysis and **(D)** Leaf disk chlorophyll assay of WT and T_1_ transgenic lines at different NaCl concentrations.

**FIGURE 3:**
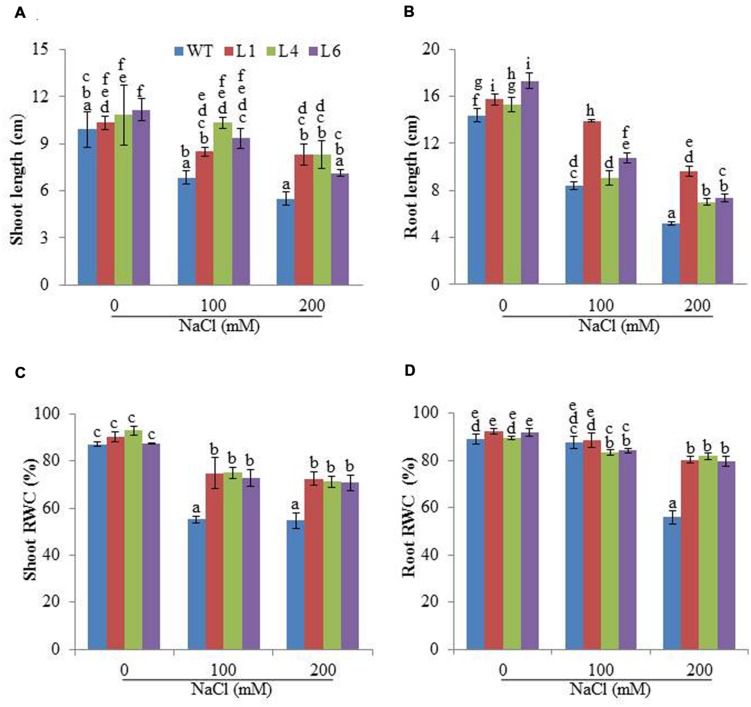
**Physiological changes in *JcPR-10a* transgenic plants grown in the presence of 0, 100, and 200 mM NaCl.** **(A,B)** Shoot and root length, **(C,D)** Relative water content (RWC) of shoot and root. Values represented are means ± SE (*n* = 3) and marked with different alphabets to indicate significant difference at *P* ≤ 0.05 probability.

The WT and transgenic (L1, L4, and L6) seeds were germinated on MS medium supplemented with 0, 100, 200, and 300 mM NaCl. Under 100 and 200 mM salt concentration, the transgenic seeds showed faster and higher percent germination as compared to WT seeds. Upon exposure to higher salt stress (300 mM), the transgenic seeds exhibited weak germination efficiency, whereas, WT seeds almost failed to germinate (**Figures 2A,B**). Twenty one days old T_1_ seedlings were transferred to hydroponic medium containing 0, 100 and 200 mM NaCl. With salinity stress, the T_1_ transgenic lines exhibited salt tolerant phenotype (**Figure 2C**) with significant enhancements in shoot and root length relative to WT plants (**Figures 3A,B**). Leaf disks of WT and T_1_ (L1, L4, and L6) lines were incubated in different concentration of salt for 3 days showed higher amount of chlorophyll in transgenic lines (**Figure 2D**). The RWC of transgenics was observed to be significantly higher in shoots at both 100 and 200 mM NaCl, whereas, in root RWC was found significantly higher only at 200 mM NaCl as compared to WT (**Figures 3C,D**).

### Physiological and Biochemical Response of *JcPR-10a* Transgenics in Response to Salinity Stress

**FIGURE 4:**
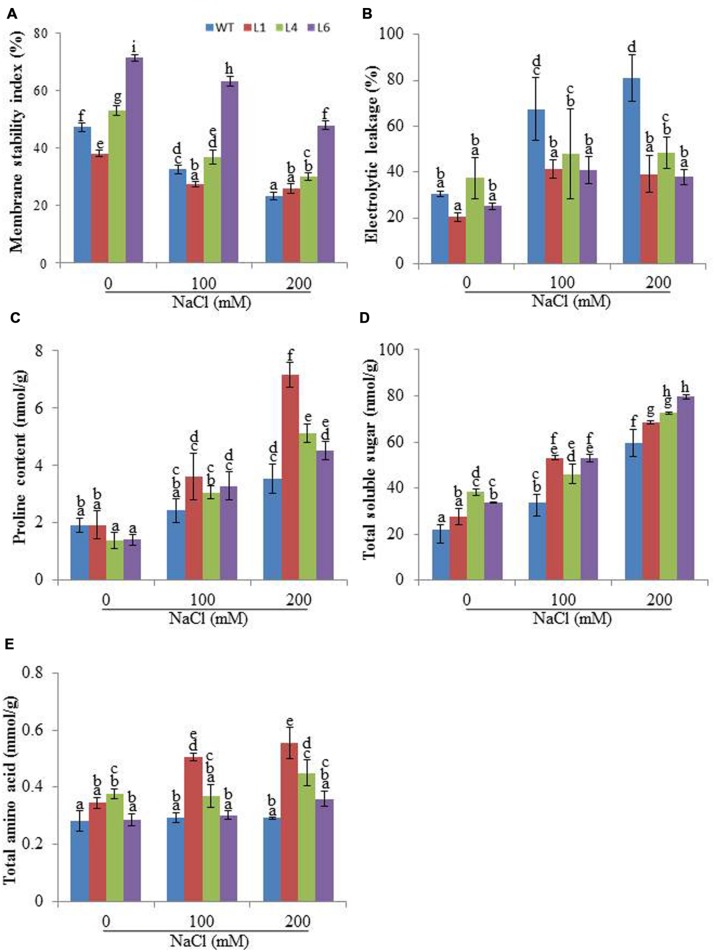
**Biochemical changes in WT and *JcPR-10a* transgenic plants grown in the presence of 0, 100, and 200 mM NaCl.** **(A)** Membrane stability index (MSI), **(B)** Electrolyte leakage (EL), **(C)** Proline content, **(D)** Total soluble sugar (TSS) content, and **(E)** Total amino acids (TAAs) content. Values are represented as means ± SE (*n* = 3) and marked with different alphabets to indicate significant difference at *P* ≤ 0.05 probability.

Membrane stability index analysis revealed that the cell membrane of *JcPR-10a* T_1_ transgenics (L1, L4 and L6) was more stable than WT, in presence of 200 mM NaCl (**Figure 4A**). Relative to WT plants, the transgenic lines exhibited significantly reduced EL during salt stress (**Figure 4B**). Proline, TSS and amino acid contents were measured in WT and transgenic plants to substantiate the function of the *JcPR-10a* gene. In the presence of 200 mM NaCl, the transgenic plants had significantly higher proline content relative to WT (**Figure 4C**). Transgenic lines and WT both exhibited a gradual accumulation of TSS by increasing NaCl concentration; however, transgenic lines exhibited significantly higher accumulation at 200 mM NaCl as compared to WT (**Figure 4D**). Similarly, at 200 mM NaCl, all the transgenics showed significantly higher accumulation of TAA compared to WT (**Figure 4E**).

**FIGURE 5:**
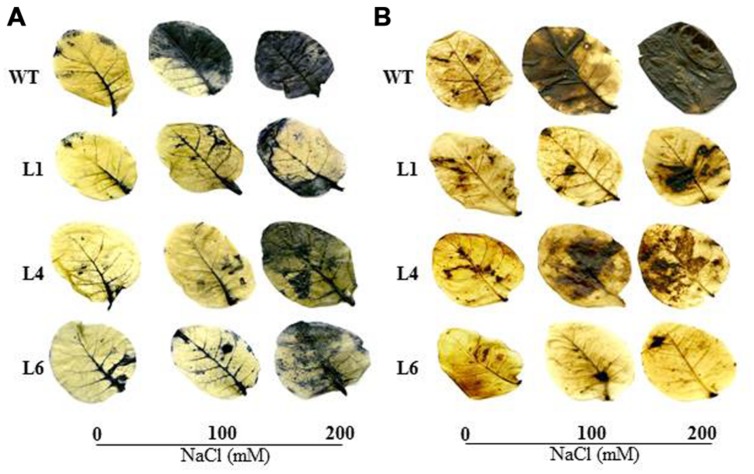
***In vivo* localization of O_2_^-^ and H_2_O_2_ in leaves of WT and transgenic lines grown in hydroponic medium.** **(A)** Localization of O_2_^-^ by NBT staining, **(B)** Localization of H_2_O_2_ by DAB staining in the presence of 0, 100, and 200 mM NaCl.

The WT and transgenics exhibited similar staining in presence of NBT and DAB at 0 mM NaCl. However, at 100 and 200 mM NaCl, WT and VA leaves accumulated more blue (indicator of O_2_^-^) and brown colored spots (indicator of H_2_O_2_) in comparison to transgenics (**Figures 5A,B**).

**FIGURE 6:**
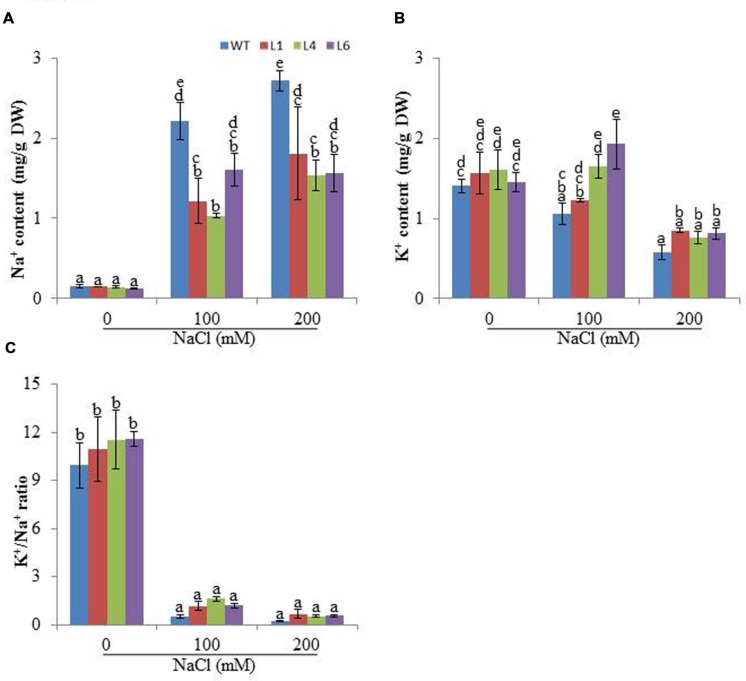
**Analysis of ion content in leaves of WT and *JcPR-10a* transgenic tobacco plants grown at 0, 100, and 200 mM NaCl, **(A)** Na^+^ content, **(B)** K^+^ content, **(C)** K^+^/Na^+^.** Values are represented as means ± SE (*n* = 3) and marked with different alphabets to indicate significant difference at *P* ≤ 0.05 probability.

Accumulation of different ions was analyzed in the presence of salinity stress. At 0 mM NaCl, Na^+^ content was approximately same in WT and transgenics. In the presence of 100 and 200 mM NaCl, all plants showed enhanced accumulation of Na^+^ ions, however, the transgenics accumulated significantly lower Na^+^ content than WT (**Figure 6A**). The K^+^ content was higher in transgenic lines when exposed to 100 and 200 mM NaCl (**Figure 6B**) and thus helped to maintain higher K^+^/Na^+^ (**Figure 6C**).

### *JcPR-10a* Transgenics Exhibit Enhanced Endogenous Cytokinin

**FIGURE 7:**
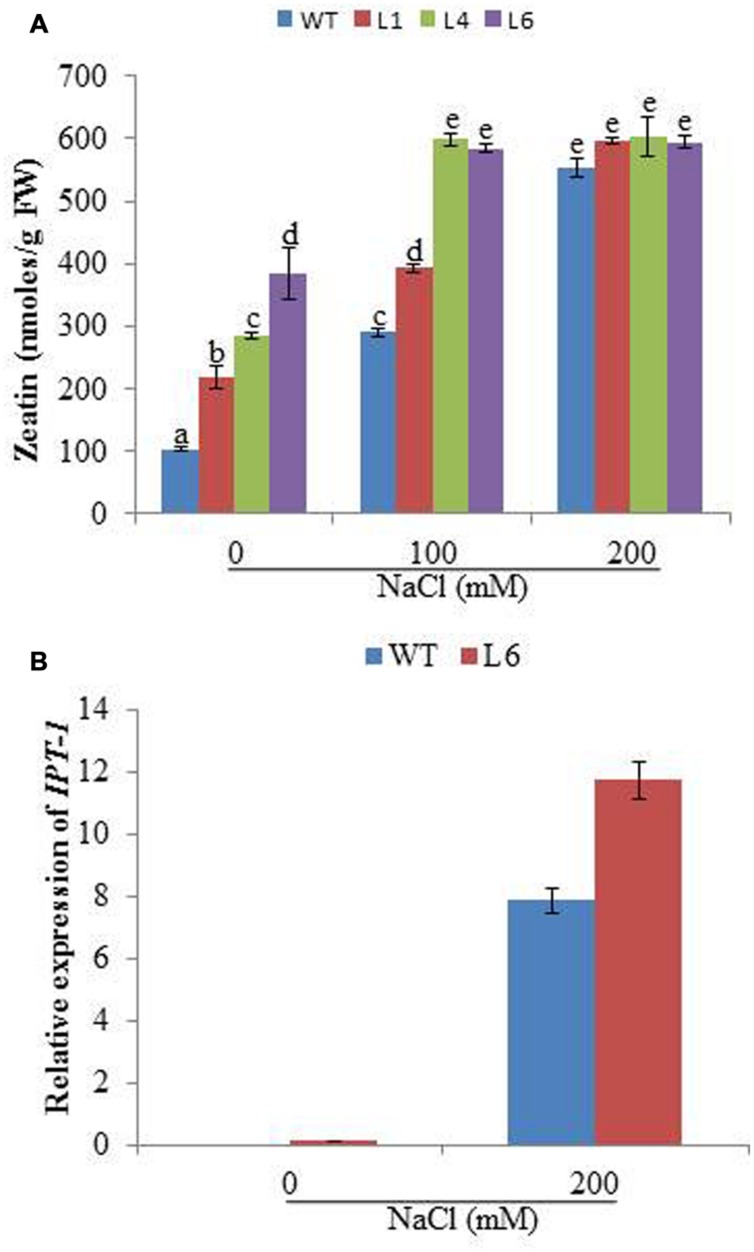
**(A)** The zeatin concentration in WT and *JcPR-10a* transgenic lines (L1, L4, and L6) at different NaCl concentrations. Data represent the mean ± SE of three biological repeats. **(B)** Relative-fold expression of *IPT-1* gene in WT and L6 transgenic line at 0 and 200 mM NaCl concentrations. Data represent the mean ± SE of three biological repeats.

The cytokinin content was studied for WT and transgenics on different concentrations of NaCl. The zeatin increased with increasing salt concentration in both WT and transgenics. At 0 mM NaCl, zeatin endogenous level in WT was 104.01 nmoles/g FW, whereas for L1, L4, and L6 it was 217.62, 284.78, and 384.34 nmoles/g FW, respectively. At 100 mM NaCl the zeatin level increased to 290 nmoles/g FW in WT, whereas in lines L4 and L6 the level increased to 598 and 584 nmoles/g FW, respectively. With 200 mM salinity stress, the WT and transgenics showed similar zeatin level (**Figure 7A**). Further the expression of cytokinin biosynthesis gene (*IPT-1*, isopentyltransferase-1) was studied in line L6 for correlating reason of high accumulation of hormones under stress condition. The line L6 showed 11.8-fold relative expression for *IPT-1* at 200 mM NaCl concentration (**Figure 7B**).

### *JcPR-10a* Transgenics Maintain Better Photosynthesis Machinery Under Salinity Stress

**FIGURE 8:**
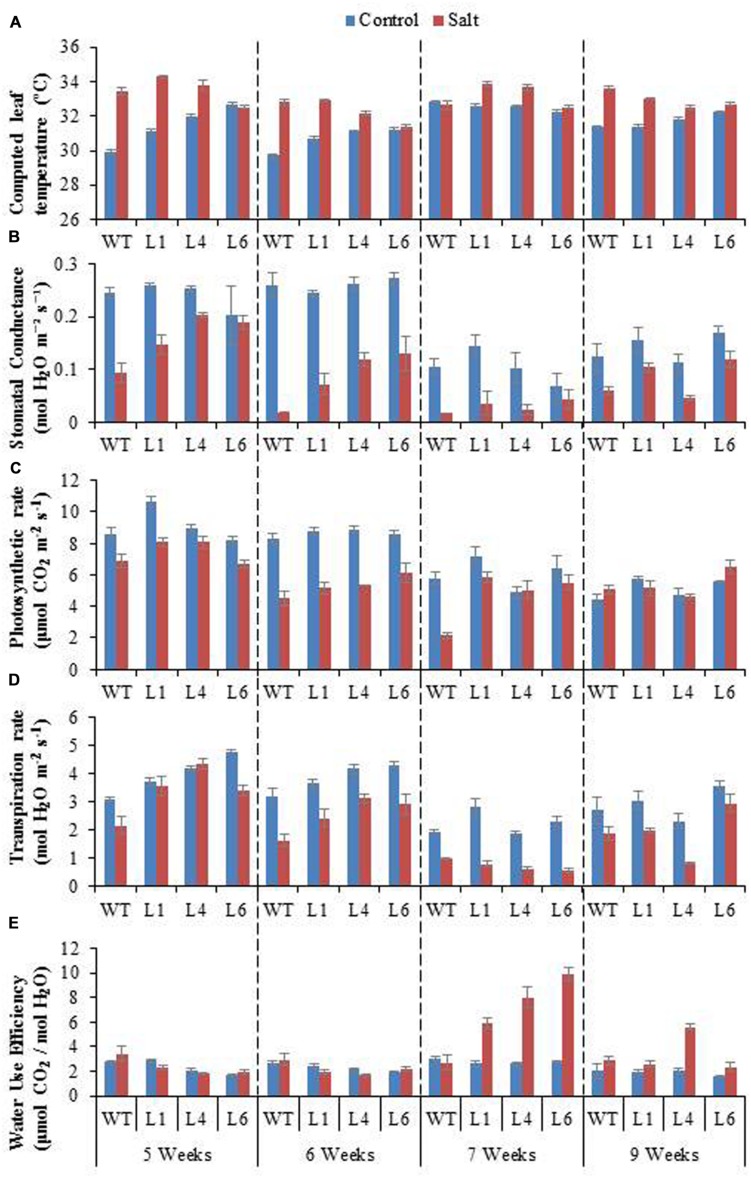
**Physiological analysis of WT and *JcPR-10a* transgenic lines (L1, L4, and L6) in response to the gradual exposure of stress (5–7 weeks) and after restoration (9 weeks).** **(A)** Computed leaf temperature, **(B)** Stomatal conductance, **(C)** Photosynthetic rate, **(D)** Transpiration rate and **(E)** Water use efficiency (WUE).

To access the effect of salinity on the photosynthetic machinery of WT and *JcPR-10a* transgenics, plants were subjected to gradual NaCl stress treatment. The leaf temperature of WT and transgenics was higher under stress conditions as compared to unstressed conditions (**Figure 8A**). The WT and transgenics exhibited similar stomatal conductance (**Figure 8B**) and photosynthesis rate (**Figure 8C**) under control conditions, however, on exposure to salinity stress, the transgenics exhibited higher stomatal conductance and photosynthesis (∼2.5-fold) as compared to WT. The extent of stomatal conductance and photosynthesis decreases with increasing concentration of NaCl and starts to enhance when plants are re-watered. The transpiration rate of transgenics was 1.3-fold higher than WT under unstressed conditions. At 300 mM NaCl, the transgenics had 0.6-fold lower transpiration rate as compared to WT (**Figure 8D**). The water use efficiency (WUE) of transgenics was ∼2.9-fold higher than WT at 300 mM NaCl (**Figure 8E**).

**FIGURE 9:**
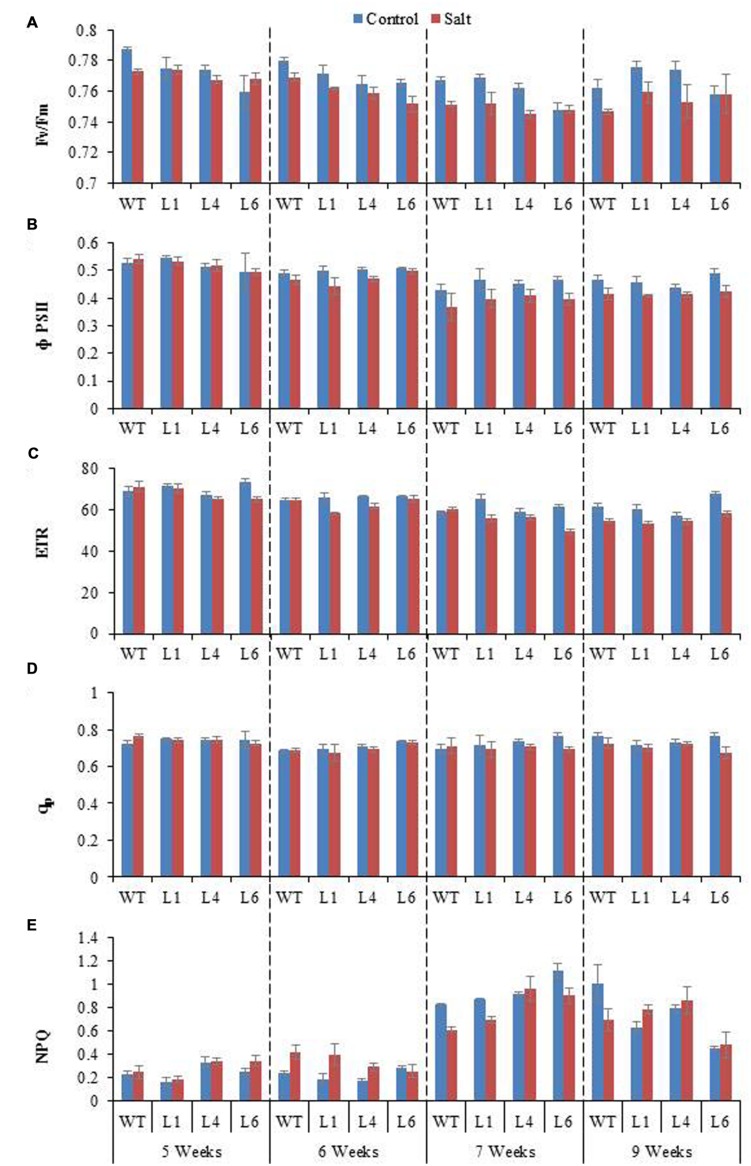
**Chlorophyll fluorescence analysis of WT and *JcPR-10a* transgenic lines (L1, L4, and L6) in response to the gradual exposure of stress from 5 to 7 weeks and on growth restoration by removal of stress (9 weeks).** **(A)** *F*_v_/*F*_m_ ratio, **(B)** Φ PSII, **(C)** Photosynthetic electron transport rate (ETR), **(D)** Photochemical quenching (q_p_) and **(E)** non-photochemical quenching (NPQ).

Chlorophyll fluorescence measurement is also an important parameter to study the physiology of plants under salinity stress. The *F*v/*F*m ratio, ΦPSII, ETR, q_p_, and NPQ of WT and transgenics was lower during salt stress treatments, and the transgenics showed better resurrection on re-watering (**Figures 9A–E**).

**FIGURE 10:**
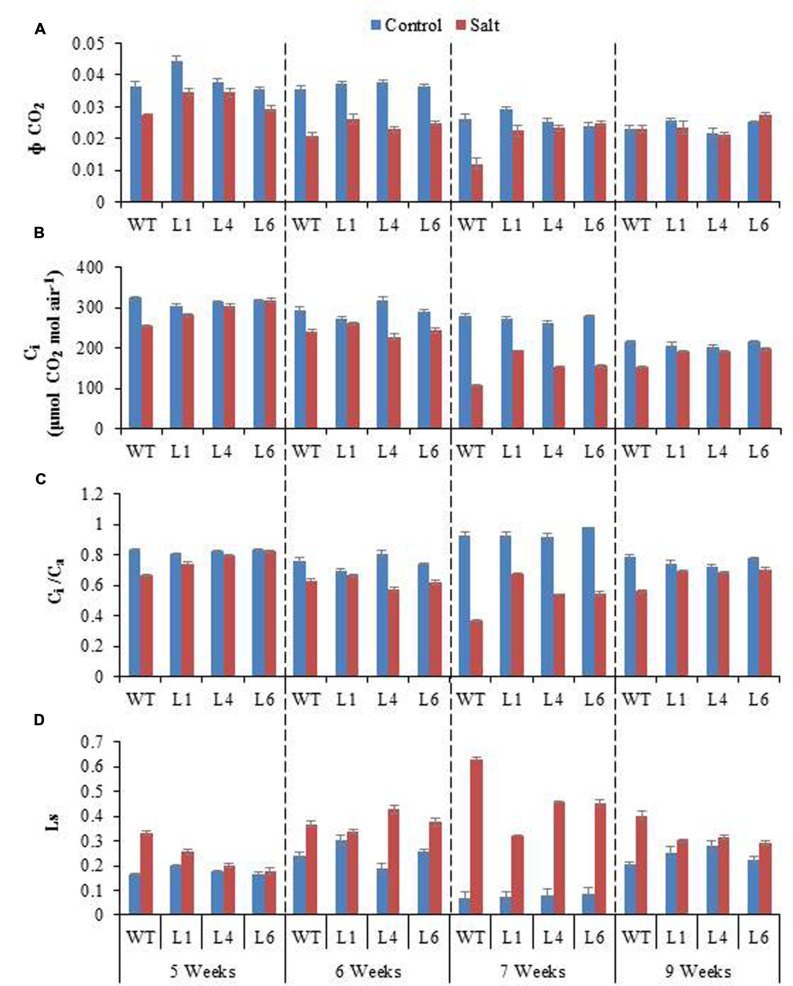
**(A)** Comparison of maximum quantum yield of CO_2_ assimilation (Φ CO_2_), **(B)** Intercellular CO_2_ concentration (*C*i), **(C)** Ratio of intercellular to ambient CO_2_ concentration (*C*i*/C*a), and **(D)** Stomatal limitation value (Ls) of WT and transgenic lines (L1, L4, and L6) in response to the gradual exposure of stress from 5 to 7 weeks and after restoration (9 weeks).

Salinity distinctly reduced the CO_2_ assimilation (Φ CO_2_), *C*i and *C*i/*C*a ratio (**Figures 10A–C**). The Φ CO_2_ declined markedly with increasing salinity, however, the transgenics showed improved Φ CO_2_ (1.9-fold) as compared to the WT plants, in the presence of 300 mM NaCl (**Figure 10A**). Salinity reduced the *C*i/*C*a ratio from 0.93 (control conditions) to 0.36 (300 mM NaCl) in WT, however, the reduction in transgenics was less, being 0.93–0.68, 0.92–0.54, 0.98–0.55 in transgenic lines L1, L4, L6, respectively (**Figure 10C**). The stomatal limitation value (Ls) is 1.5-fold higher in WT as compared to transgenic lines, when plants are exposed to 300 mM NaCl (**Figure 10D**). After recovery from stress the *C*i/*C*a improves and the value of Ls gets lowered in both WT and transgenic lines, however, the recovery process is quick and better in transgenics (**Figures 10C,D**).

**FIGURE 11:**
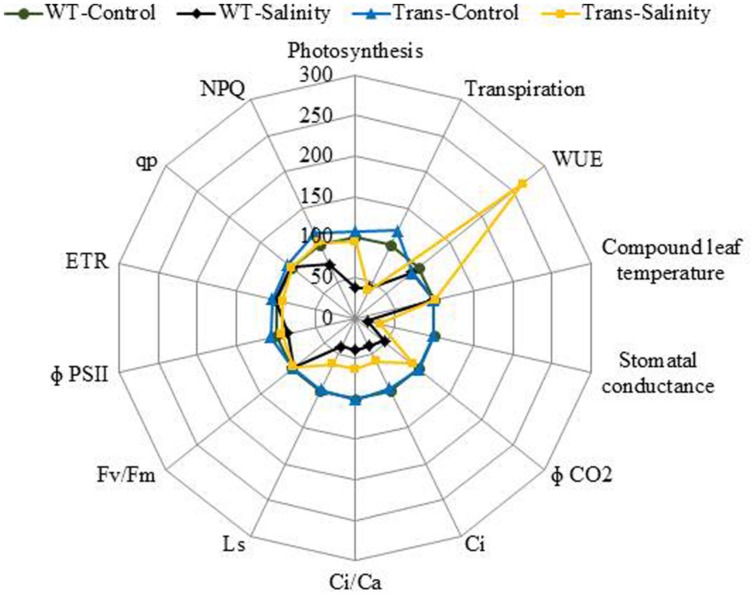
**The radar diagram built by comparing all the photosynthesis related parameters between WT and transgenic plants (average of three transgenic lines) under control and 300 mM NaCl treatment**.

The radar diagram was built by comparing all the photosynthesis related parameters between WT and transgenic plants under control and 300 mM salinity treatment (**Figure 11**). Both the WT and transgenic plants showed similar behavior under control condition but at 300 mM salinity, wild type and transgenic plants have different behavior. The wild type plants under salinity showed noticeably lower photosynthesis, stomatal conductance, Φ CO_2_, *C*_i_, *C*_i_/*C*_a_, Ls and NPQ as compared to control plants (**Figure 11**). On the other hand, transgenic plants under salinity showed lower transpiration, stomatal conductance, *C*_i_, *C*_i_/*C*_a_, Ls but same photosynthesis, Φ CO_2_ and NPQ as compared to control plants. Under salinity condition, transgenic plants showed higher WUE as compared to control plants. Some parameters like computed leaf temperature, *F*v/*F*m, Φ PSII, ETR, and q_p_ didn’t show any variations in WT and transgenic plants under both control and saline condition (**Figure 11**).

### *JcPR-10a* Transgenics Exhibit Antifungal Activity to *Macrophomina phaseolina*

**FIGURE 12:**
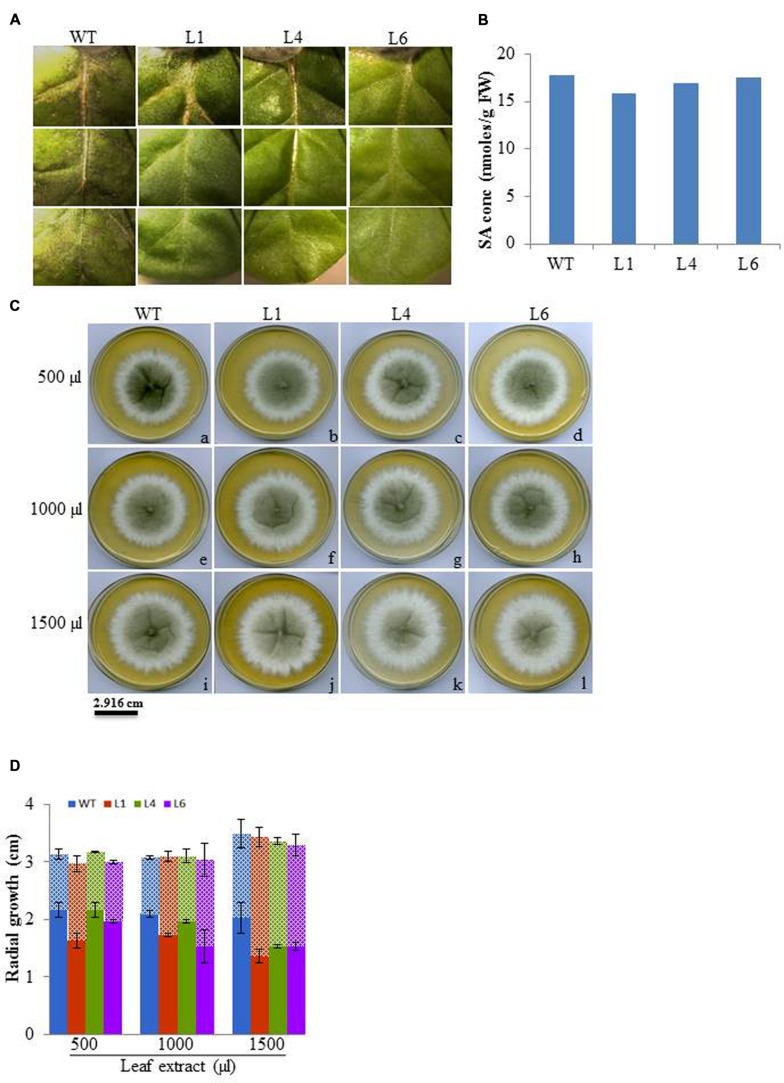
**(A)** Severity of infection on WT and *JcPR-10a* transgenics (L1, L4, and L6) leaf incubated with *Macrophomina phaseolina*. **(B)** Salicylic acid content of healthy WT and transgenic (L1, L4, and L6) leaves. **(C)** Differential growth of *Macrophomina* fungus in potato dextrose medium supplemented with different amount (500, 1000, and 5000 μl) of leaf extract from WT and transgenic lines (L1, L4, and L6). The black and white zone represents the reproductive and vegetative growth, respectively. **(D)** Graph representing the reproductive (microspores, in bold column) and vegetative (hypha, in pattern) growth. Data represent the mean ± SE of three biological repeats.

The leaf bioassay revealed that WT leaves challenged with *M. phaseolina* inoculum along the detached mid vein region developed severe infection all along the midrib region from the proximal to distal end of the leaf as soon as 3 dpi, whereas, the transgenics showed almost negligible infection (**Figure 12A**). The similar WT and transgenic leaf were used for SA quantification, and it was observed that both WT and transgenic leaf showed same SA content (**Figure 12B**). Anticipating that the tobacco transgenics overexpressing *JcPR-10a* gene might be exhibiting antimicrobial activity, the antifungal activity of crude leaf extracts of WT and transgenic lines was studied. In the leaf extract assay, the intensity of microsporangia formation is greatly reduced as is evident by the decrease in the black colouration with increasing concentration of the transgenic leaf extract (**Figure 12C**). The analysis of radial growth of *Macrophomina*, revealed that at 500 μl concentrations, only L1 shows significantly lower reproductive growth, whereas at 1500 μl concentration significantly lower growth of approximately 0.7-fold is observed with all the transgenics as compared to WT (**Figure 12D**).

## Discussion

The PR-10 proteins are interesting multifunctional proteins playing an important role in plants response to intrinsic and extrinsic factors, however, the signaling pathway involved in its activation remains unclear. PR-10 gene family has been identified in wide variety of plant species and show low- and higher-interspecific variations (Wen et al., 1997; Lebel et al., 2010). The wide distribution throughout the plant kingdom, highlight their indispensable function in plants, however, their true biological role remains obscure. The major role of PR-10 proteins is reported in response to biotic and abiotic stresses.

The *JcPR-10a* transformed explants showed higher number of shoot bud induction and well differentiated shoots on regeneration medium as compared to vector alone. The higher shoot bud induction could be attributed to the increased cytokinin content in the transgenics as evident by HPLC analysis. The cytokinins play a pivotal role in shoot organogenesis (Hill and Schaller, 2013). The cytokinin and auxin hormones *in vivo* regulate cell division, differentiation and meristem establishment. The *JcPR-10a* overexpression lines maintain higher endogenous cytokinin/auxin ratio, therefore the explants showed higher shoot regeneration with no callusing even on low concentration of BAP. This phytohormone ratio play an important role in plant tissue culture development, these hormones have antagonistic as well as synergistic roles (Skoog and Miller, 1957). The variations in cytokinin to auxin ratios favor development of either shoot or root meristems (Sugimoto et al., 2011).

The PR-10 proteins show general ligand binding, where both PR-10 and ligand show mutual conformational changes allowing transport of ligands from cytosol to their receptors (Fernandes et al., 2008, 2009, 2013). The binding of PR-10 proteins with different physiological ligands including cytokinin, flavonoids and fatty acids has been reported (Mogensen et al., 2002). In our study, the 3D-ligand binding software (Wass et al., 2010) predicted the presence of cytokinin binding sites in the JcPR-10a protein. Further, the docking studies of JcPR-10a protein explicitly showed that three molecules of BAP bind at its active sites. This confirms that JcPR-10a protein has affinity with BAP, which may be the cause of high shoot regeneration in the transgenic lines. Fernandes et al. (2009) reported the structural adaptability of LlPR-10.2B protein to bind to different cytokinins suggesting that PR-10 proteins might serve as a cytokinin reservoir in the plant cell. The cytokinin-specific binding protein from mung bean (VrCSBP) and intracellular PR protein from moss have also shown cytokinin binding activity (Fujimoto et al., 1998; Gonneau et al., 2001). The putative 3D structure of JcPR-10a protein, shows similarity to the other Betv1 protein structures (Agarwal et al., 2013). The Betv1 contain the polyketide cyclase domain involved in binding/transport of lipids and in the synthesis of compounds like pigments, antibiotics and anti-tumor drugs. The polyketide cyclase/dehydrase-like domain of Betv1 is ubiquitous domain, involved in binding of large hydrophobic ligands (Radauer et al., 2008). The yellow lupine PR-10 protein (LlPR-10.2B) showed binding to trans-zeatin, interestingly; similar ligand binding has also been reported for plant cytokinin-specific binding proteins (CSBP, Pasternak et al., 2006).

The *JcPR-10a* transgenics showed higher growth and productivity under salinity by enduring better morphological growth. Similarly, the over expression of potato *PR-10a* (formerly *STH-2*) gene significantly enhanced salt and osmotic stress tolerance in transgenic potato suspension cultures (El-bannaa et al., 2010). Constitutive expression of pea *PR-10.1* showed enhanced seed germination of *Brassica napus* under saline conditions (Srivastava et al., 2004). Recently, Jain et al. (2012) showed that overexpression of *AhSIPR10* gene from peanut enhanced abiotic stress tolerance by reducing ionic and oxidative stress in transgenic plants exposed to salinity, heavy metal or drought stress. The enhanced tolerance of the *JcPR-10a* transgenic could be attributed to increased RWC, higher MSI, reduced EL, ionic accumulation and oxidative damage. The enhanced abiotic stress tolerance could be achieved by higher level of endogenous cytokinin in *JcPR-10a* transgenics, which is further increased on exposure to salt stress and might be involved in mitigating the salinity-induced damage. The increased cytokinin levels enhanced the resistance against salinity by functioning as antioxidants (Gidrol et al., 1994). Increased cytokinin levels maintain high cellular redox potential during drought and hence reduce damage by ROS (Rivero et al., 2007). Zwack and Rashotte (2015), mentioned the complex role of cytokinin in abiotic stress responses, suggesting that the cytokinin concentrations show a transient increase on initial stress, followed by maintenance of increased cytokinin concentrations with increased stress conditions. Similarly, the *JcPR-10a* transgenics showed an increased level of endogenous cytokinin from 0 to 100 mM NaCl stress as compared to WT and further maintained almost similar concentration of cytokinins at 200 mM NaCl in transgenic plants (L4 and L6). Although at 200 mM, the WT and transgenics exhibited similar cytokinin concentration but there exists variation in the transcript of the *IPT-1* gene. In L6, at 200 mM NaCl, a higher *IPT-1* expression was observed as compared to WT plants.

Photosynthesis is a key phenomenon, during which light energy is converted into utilizable form of chemical energy by all green plants for their growth and development. Salinity stress imposes both hyper osmotic stress as well as hyper ionic stress (Chaves et al., 2009). The salinity, therefore, regulates photosynthesis either directly causing diffusion limitations through stomata of mesophyll (Flexas et al., 2007) or through alterations in photosynthesis metabolism (Lawlor and Cornic, 2002) or indirectly by the generations of oxidative stress (Chaves and Oliveira, 2004). The efficacy of WT and transgenics was analyzed by gradual NaCl stress, as Shavrukov (2013) recommend that imposition of gradual salinity treatments reflects the natural condition in which salinity exists in ecosystems. The NaCl stress was given from 5 to 7 weeks with 100, 200, 300 mM NaCl, respectively. The transgenics exhibited lower transpiration rate and higher leaf temperature, stomatal conductance, photosynthesis rate and WUE as compared to WT during 7th week at maximum NaCl stress. Salinity impacted the photosynthesis of both WT and transgenics by reducing stomatal conductance, leading to decreased diffusion of CO_2_ to the carboxylation sites. The overexpression of the *AhSIPR10* gene in tobacco showed increased photosynthesis during abiotic stress (Jain et al., 2012). A low transpiration rate reduces the salt loading into the leaves, especially the juvenile ones, and thus maintains salts at subtoxic levels (Koyro, 2006). The enhanced photosynthesis and reduced transpiration rate in transgenics with severe salinity led consequently to a significant increase in WUE. The WUE, is measured as the biomass produced per unit transpiration, and shows the relationship between water use and crop production, therefore it is an important parameter for sustained agriculture practice. The basic physiological definition of WUE is the ratio of photosynthesis to transpiration, also referred to as transpiration efficiency. Forward and reverse genetic approaches are being used to discover and study genes that improve WUE in major crop species. The *ERECTA, HARDY*, and *DREB1A* gene from *Arabidopsis* enhanced transpiration efficiency in *Arabidopsis*, rice and peanut, respectively (Masle et al., 2005; Karaba et al., 2007; Sarkar et al., 2014). Also, the *HVA1* gene of barley improved WUE in wheat (Sivamani et al., 2000).

Baker (2008) reported that Fv/Fm ratio reflects the whole PSII function, and its decrease indicates the PSII damage or photoinhibition under environmental stress. The PSII function has been shown to be sensitive as well as resistant to salt stress (Chen et al., 2004; Netondo et al., 2004; Yan et al., 2012). The NPQ measures the photo protective heat dissipation of the PSII antenna complexes (Gilmore, 1997). The decrease in PSII with an increase in NPQ with no subsequent decline in *F*v/*F*m at high salinity level maintain PSII function, thereby only slight inhibition of photosynthesis occurred at high salt level in the *JcPR-10a* transgenics. All the photosynthesis related parameters showed maximum changes at 300 mM NaCl treatment. Under saline conditions WT plants showed poor photosynthesis but transgenic plants showed no change in photosynthesis compared to control. This might be because of the increased WUE in transgenic plants under salinity that helped the plant to maintain its photosynthetic activity even under high salinity stress. All these data showed that the *JcPR-10a* transgenic plants are more tolerant to high salinity stress and can maintain their physiology even under stressed conditions.

The enhanced endogenous cytokinin level of transgenics under control as well as stressed condition might also be protecting the photosynthetic machinery. Interestingly, Cortleven and Valcke (2012) reported that both an increase as well as a decrease in cytokinin content results in a better photosynthetic performance, as cytokinins can induce changes in the kinetics of the electron transfer reactions in PSII during photosynthesis. The proteomic study on photosynthetic parameters of transgenic tobacco with altered cytokinin content, reveal few significant differences in the stroma proteins involved in Calvin-Benson cycle and photoprotective mechanisms against oxidative damage (Cortleven et al., 2011).

The fungal resistance assays of WT and *JcPR-10a* transgenics elucidated the potential role of JcPR-10a in enhancing resistance against *Macrophomina.* In the leaf assay, 3 dpi of *Macrophomina* showed severe blackening along the mid vein and side veins from proximal to distal end of the WT leaf only. Furthermore, in the leaf extract assay the radial growth and intensity of microsclerotia is greatly inhibited with the transgenic leaf extract, indicating that the JcPR-10a protein shows antifungal activity. This could be due to induced RNase activity. Park et al. (2004) have reported that CaPR-10 protein’s RNase activity cleaves viral RNA. Earlier, we have shown that JcPR-10a has both RNase and antifungal activity, whereas boiled protein lacked RNase and antimicrobial activity (Agarwal et al., 2013). Similarly, TcPR-10 protein showed both RNase and antifungal activity, and its denaturation led to loss of activity and inability to be taken by fungal cells (Pungartnik et al., 2009). Argueso et al. (2012) provided a model involving complex crosstalk between cytokinin and SA in plant immunity, a mechanism involving two-component signaling elements, during which cytokinin up-regulates plant immunity by promoting the SA-dependent defense responses. The *JcPR-10a* transgenics did not show enhanced SA concentrations under normal growth conditions but it might get enhanced after induction of biotic stress. At this point, the enhanced SA might be helping the plants to develop biotic stress tolerance through increased RNase activity, by programmed cell death (PCD) at and around the plant infection sites (Liu and Ekramoddoullah, 2006). Zubini et al. (2009) showed a correlation between RNase hydrolysis and cytokinin binding; on incubation of Pru p 1.01 recombinant protein with zeatin, the RNase activity was inhibited for 1 h and later restored. This correlation suggested a mechanism of alternate zeatin/RNA binding to protein for its specific functioning.

## Conclusion

Our study revealed that the enhanced regeneration potential of the transgenics could be attributed to high affinity of the JcPR-10a protein to cytokinin. The enhanced endogenous cytokinin level in transgenics regulated different biochemical and molecular parameters leading to enhanced salinity tolerance. The transgenics exhibited reduced oxidative damage with enhanced WUE and photosynthesis during salinity stress. The transgenics plants showed resistance toward *M. phaseolina* infection possibly through involvement of its RNase activity. These results highlight the broad spectrum role of *JcPR-10a* gene *in planta*, in regulating growth-development and overcoming stress, and thereby, can be deployed for sustained growth and productivity of plants during multiple stresses.

## Author Contributions

PA carried out gene cloning, molecular analysis, experiment execution and designing, data analysis and manuscript writing. MD carried out genetic transformation and biochemical experiments. PM involved in photosynthesis experiment. KP involved in hormone and expression analysis. KJ carried out docking experiment. PKA coordinated all the experiments and MS writing. All authors approved the final manuscript.

## Conflict of Interest Statement

The authors declare that the research was conducted in the absence of any commercial or financial relationships that could be construed as a potential conflict of interest.

## Supplementary Material

**FIGURE S1 | Characterisation of transgenic tobacco plants.** **(A)** Schematic representation of the pCAMBIA1301 – 35S: JcPR-10a construct used for *Nicotiana tabacum* transformation. **(B)** GUS assay of T_0_ leaf and T_1_ seedlings showing positive expression in the transgenic lines and negative expression in WT plants. PCR confirmation of transgenic lines with **(C)** GUS, **(D)** *hptII*, and **(E)** *JcPR-10a* primers. **(F)** GUS:NRA ratio for determination of copy number of transgene insertion in transgenics.

**Table S1 | List of primers used in this study.**

**Table S2 | Segregation ratio of transgenics.**
