# Supplementary figures and images for: Improved Shoot Regeneration, Salinity Tolerance and Reduced Fungal Susceptibility in Transgenic Tobacco Constitutively Expressing PR-10a Gene
Source: Front Plant Sci. 2016 Feb 29;7:217. doi: 10.3389/fpls.2016.00217 (PMC4770195; doi:10.3389/fpls.2016.00217)

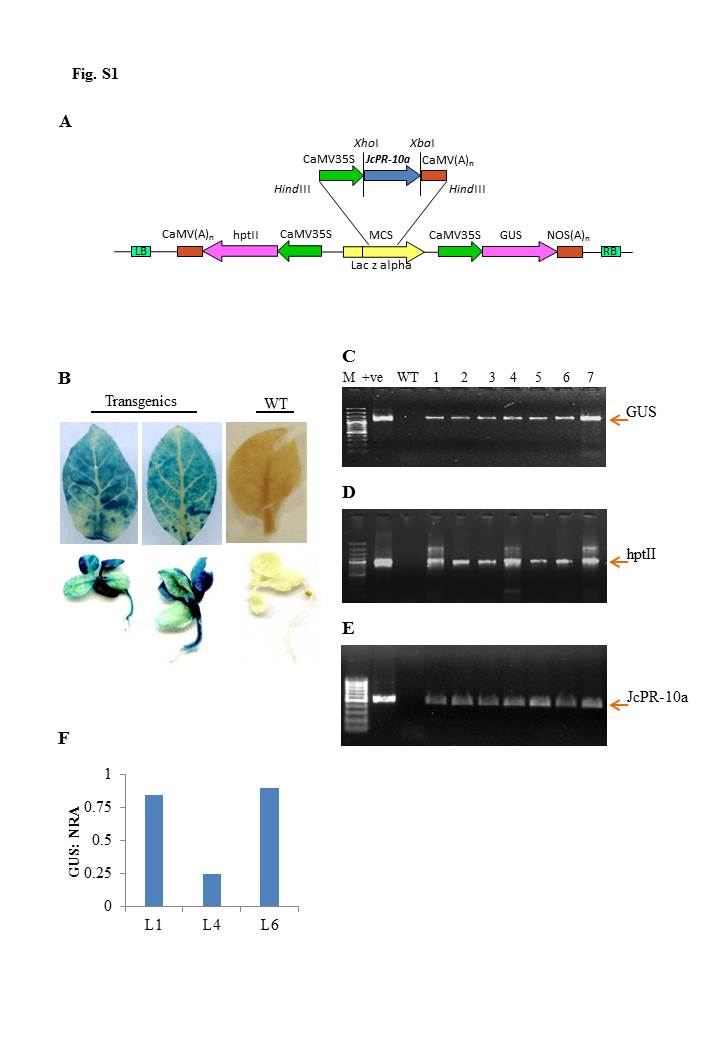

Supplement: Supplementary file 1 [file Image_1.JPEG]
