# Supplementary material for: Improved Shoot Regeneration, Salinity Tolerance and Reduced Fungal Susceptibility in Transgenic Tobacco Constitutively Expressing PR-10a Gene
Source: Front Plant Sci. 2016 Feb 29;7:217. doi: 10.3389/fpls.2016.00217 (PMC4770195; doi:10.3389/fpls.2016.00217)
Supplement: Supplementary file 2 [file Table_1.DOCX]

**Table S1** List of the primers used in this study

| **Primer ID** | | **Primer Sequence** | |  |  |
| --- | --- | --- | --- | --- | --- |
| JcPR10a TF  JcPR10a TR  hptII F  hptII R  GusA F  GusA R  GQF  GQR  NRA F  NRA R  NtActin F  NtActin R  NbIPT1F  NbIPT1R | | 5’-GCTCGAGATGGCTGTTACTGTCTTT-3’  5’-TGCTCTAGATTAGTAGGCATTAGGATT-3'  5’-TTCTTTGCCCTCGGACGAGTG-3′  5′-ACAGCGTCTCCGACCTGATG-3′  5’-GATCGCGAAAACTGTGGAAT-3’  5’-TGAGCGTCGCAGAACATTAC-3’  5’-GTGAAGGGCCAACAGTTCC-3’  5’-GGTAATGCGAGGTACGGTA-3’  5’-AATGCTGGCACTGATTGCAC-3’  5’-TCCTCTGCGCTGGAACAAG-3′  5’-GATTTGCTGGTGATGATGCTCC-3’  5’-GTCTCAAACATGATCTGTGTCATC-3’  5’-AAGCTTTCTATCGATCTCCGCCAC-3’  5’-CAATACAGTGCCCAAAACATC-3’ | |  |  |
|  | |  | |  | |
