# Supplementary material for: Improved Shoot Regeneration, Salinity Tolerance and Reduced Fungal Susceptibility in Transgenic Tobacco Constitutively Expressing PR-10a Gene
Source: Front Plant Sci. 2016 Feb 29;7:217. doi: 10.3389/fpls.2016.00217 (PMC4770195; doi:10.3389/fpls.2016.00217)
Supplement: Supplementary file 3 [file Table_2.DOCX]

**Table S2** Segregation ratio of transgenics

| **Transgenic lines** | **No of seeds inoculated** | **No. of seeds germinated** | **Ratio** **HYG^r^ : HYG^s^** |
| --- | --- | --- | --- |
| L1 | 554 | 428 | 3.396 |
| L4 | 591 | 459 | 3.477 |
| L6 | 663 | 498 | 3.018 |
| VA | 456 | 350 | 3.301 |
